# Single-nuclei RNA-seq reveals skin cell responses to *Aeromonas hydrophila* infection in Chinese longsnout catfish *Leiocassis longirostris*

**DOI:** 10.3389/fimmu.2023.1271466

**Published:** 2023-10-16

**Authors:** Cheng-Yan Mou, Lu Zhang, Han Zhao, Zhi-Peng Huang, Yuan-Liang Duan, Zhong-Meng Zhao, Hong-Yu Ke, Jun Du, Qiang Li, Jian Zhou

**Affiliations:** Fisheries Research Institute, Sichuan Academy of Agricultural Sciences, Chengdu, Sichuan, China

**Keywords:** single nuclei RNA-sequencing, fish skin cell, immune response, *Aeromonas hydrophila*, cell expansion

## Abstract

As the primary natural barrier that protects against adverse environmental conditions, the skin plays a crucial role in the innate immune response of fish, particularly in relation to bacterial infections. However, due to the diverse functionality and intricate anatomical and cellular composition of the skin, deciphering the immune response of the host is a challenging task. In this study, single nuclei RNA-sequencing (snRNA-seq) was performed on skin biopsies obtained from Chinese longsnout catfish (*Leiocassis longirostris*), comparing *Aeromonas hydrophila*-infected subjects to healthy control subjects. A total of 19,581 single nuclei cells were sequenced using 10x Genomics (10,400 in the control group and 9,181 in the treated group). Based on expressed unique transcriptional profiles, 33 cell clusters were identified and classified into 12 cell types including keratinocyte (KC), fibroblast (FB), endothelial cells (EC), secretory cells (SC), immune cells, smooth muscle cells (SMC), and other cells such as pericyte (PC), brush cell (BC), red blood cell (RBC), neuroendocrine cell (NDC), neuron cells (NC), and melanocyte (MC). Among these, three clusters of KCs, namely, KC1, KC2, and KC5 exhibited significant expansion after *A. hydrophila* infection. Analysis of pathway enrichment revealed that KC1 was primarily involved in environmental signal transduction, KC2 was primarily involved in endocrine function, and KC5 was primarily involved in metabolism. Finally, our findings suggest that neutrophils may play a crucial role in combating *A. hydrophila* infections. In summary, this study not only provides the first detailed comprehensive map of all cell types present in the skin of teleost fish but also sheds light on the immune response mechanism of the skin following *A. hydrophila* infection in Chinese longsnout catfish.

## Introduction

*Aeromonas hydrophila* is a significant gram-negative opportunistic pathogen that is widely distributed in aquatic environments (1). Diseases caused by *A. hydrophila* have been observed in various species of freshwater fish worldwide, resulting in considerable economic losses (2, 3). Fish infections caused by *A. hydrophila* can be transmitted to humans through animals, which is a common occurrence in cases of human-animal-fish infections (4, 5). Fish infected with *A. hydrophila* typically exhibit symptoms such as skin ulcerations and hemorrhagic septicemia (3). The skin mucosal immune system of aquatic organisms faces significant challenges as it serves as a protective barrier between the fish’s body and the surrounding water environment (6, 7).

In terms of structure, the human skin is composed of two layers: the epidermis and the dermis. The epidermis is located on the surface of the skin and can be further divided into the stratum corneum and the basal layer (8, 9). Beneath the skin is the subcutaneous tissue, which is a loose connective tissue containing a large number of fat cells (10, 11). The skin also includes various appendages such as hair, sweat glands, and sebaceous glands (12, 13). Similar to other vertebrates, the skin of fish is mainly composed of different types of epidermal and dermal layers (14). The epidermal layer is primarily composed of stratified squamous epithelial cells, mucous cells, rod-shaped cells, and basal cells that originate from the ectoderm. It forms the outermost protective barrier of the organism. The dermal layer, located beneath the epidermis, is mainly derived from the mesenchyme of the mesoderm (14–17). However, for fish, different species have different skin structures, making the study of fish skin more challenging.

In mammals, the skin is the largest organ and is in continuous contact with the environment (18). To protect the host from infection, it has developed various strategies. In addition to physical, microbiological, and chemical barriers, the skin contains resident immune cells that serve as sentinels and contribute to tissue homeostasis (19–25). When exposed to damage, these cells work together to initiate local inflammatory responses and prepare for adaptive immunity when needed (9). Therefore, one of the main functions of the skin is to protect the host from infection by pathogenic microorganisms (9). Similarly, fish skin is also considered a multifunctional tissue that serves various functions, including physical protection, sensory activities, behavioral purposes, and hormone metabolism. Additionally, it serves as the important first line of defense against pathogens for fish (26). In fish skin, a wide range of antimicrobial peptides (AMPs) can be expressed, including liver-expressed antimicrobial peptides (27), piscidins (28), and certain lipoproteins (29). These peptides typically exhibit selective activity against pathogenic bacteria, fungi, algae, viruses, or parasites (30, 31). Furthermore, research has shown that the skin of some fish develops subepidermal antibody-secreting cells similar to B lymphocytes, which are part of the fish immune system. These cells provide long-term, antigen-specific humoral immunity against reinfection by the same pathogen (32). With the rising popularity of transcriptome analysis, numerous studies have directed their focus toward exploring the molecular aspects of skin immunity by examining the transcription profile of fish skin. In species such as mud loach *Misgurnus anguillicaudatus* and sea trout *Salmo trutta*, *de novo* assembly of the skin transcriptome has unveiled potential genes associated with immunity and epidermal mucus secretion (33, 34). The skin of Atlantic cod *Gadus morhua* exhibits high expression of a significant number of genes involved in antibacterial activity and antiviral response (35). Furthermore, the skin transcriptional profiles of zebrafish *Danio rerio*, grouper (*Epinephelus coioides*), and crucian carp *Carassius auratus* from the Cyprinidae family were examined upon *A. hydrophila* infection. This analysis revealed that differentially expressed genes (DEGs) in the skin were primarily associated with immune regulation responses (1, 2, 36). Nevertheless, these findings do not offer insights into the distinctive molecular signatures that emerge when skin cells face pathogens. Moreover, due to the functional diversity, intricate anatomical structure, and complex cellular composition of the skin, it proves challenging to ascertain the particular type of cell that is targeted by the bacteria or virus, as well as unraveling the host immune response. Therefore, it is crucial to categorize the various cell types present in the skin of fish. This categorization is necessary in order to gain a comprehensive understanding of the molecular mechanisms that underlie the interaction between bacterial pathogens and the skin. Single cell RNA-sequencing (scRNA-seq) is an exceptionally potent tool for identifying target cells involved in pathogenesis. It has empowered researchers to swiftly acquire a vast amount of physiological and pathological information concerning the immune system (37–40). To the best of our knowledge, comprehensive profiling of individual cells in fish skin utilizing the latest emulsion-capture technology has not been conducted on healthy skin. Additionally, there is a lack of reported single-cell analysis on fish skin infected with bacteria.

This study presents an analysis of snRNA-seq data from 19,581 cells collected from healthy controls and *A. hydrophila*-infected Chinese longsnout catfish (*Leiocassis longirostris*) skin. By employing established cell lineage markers, a comprehensive map was constructed to identify all cell types in the skin under both conditions. Furthermore, the cellular composition and gene expression profiles were examined, and pathway enrichment analysis was conducted on different cell populations. Notably, the findings highlight the potential significance of neutrophils in defense against *A. hydrophila* infections. The datasets generated in this study serve as a valuable resource for in-depth investigations into *L. longirostris* skin and lay the groundwork for further exploration into the mechanisms of bacterial infections in teleost skin.

## Materials and methods

### Fish and bacterial infection

*A. hydrophila* was obtained from the germ bank of the Sichuan Academy of Agricultural Sciences and cultured on Todd-Hewitt agar (THA) at a temperature of 25°C with shaking at 150 rpm. The adult *L. longirostris* used in this study were 1 year old (mean weight: 60.04 ± 7.63 g, mean body length: 183.50 ± 7.77 cm). These specimens were obtained from the breeding and releasing station affiliated with the Fisheries Institute of the Sichuan Academy of Agricultural Sciences. Following a previously established testing method, five fish were subjected to a 30-minute soaking period in 5 L of 10^6^ CFU *A. hydrophila* at a temperature of 26.0 ± 1°C. Subsequently, the fish were transferred to a freshwater tank maintained at the same temperature (41). The control group was kept at the same temperature without undergoing any treatment. After 48 hours, skin samples were collected from all fish for snRNA-seq analysis.

### Single-nuclei cell capture and cDNA amplification

In this study, the nucleus was isolated from the dorsal skin cells of five control fish and five experimental fish. For the experimental group, we observed classic symptoms of *A. hydrophila* infection, such as renal hemorrhage and whitening of the liver. Blood spots also appeared on the body’s surface. In each group, non-lesional skin tissue was collected from the same location in each individual and immediately frozen for preservation. Subsequently, 50 mg of skin tissue was separated from each sample in each group and transferred to a homogenizer (Dounce) for pooling and thorough mincing. Next, 500 μL of pre-cooled cracking buffer was added to the homogenizer to extract the nuclei, following the previously described method (42). The extracted white nuclear layer was carefully washed with a nuclear cleaning buffer and then filtered through a 40 μm cell screen. Subsequently, the resulting mixture underwent a centrifugation process using an iodixanol gradient solution. Nucleus integrity was detected using trypan blue, while nucleus concentration was determined using a hemocytometer and microscope. To obtain single-nuclei gel bead-in-emulsion (GEMs), the nucleus suspensions were loaded onto the Chromium Controller instrument using the Chromium Single Cell 3’ Reagent v3 Kits (10× Genomics). The resulting cDNAs were then subjected to PCR amplification to generate an adequate mass for library construction.

### Single-nuclei RNA sequencing

The indexed sequencing libraries were constructed using the Chromium Single Cell 3’ Library v3 Kit (10x Genomics) in accordance with the manufacturer’s instructions. Subsequently, quantitative analysis of the barcoded sequencing libraries was performed using the Agilent Bioanalyzer 2100 system (Agilent, USA). Finally, the resulting libraries were sequenced on an Illumina 10× Genomics Chromium platform (10× GENOMICS) in a paired-end sequencing mode.

### Initial quality control

The original base calling files were converted into fastq data using the bcl2fastq conversion software. During the analysis of the raw data, the fastq data were aligned to the genome sequences of *L. longirostris* (accession number: PRJNA692071) using the STAR aligner with default parameters. Through the initial quality control, barcodes and UMIs that did not meet the requirements were excluded. The CellRanger count algorithm was then utilized to generate single-cell gene counts for each library (43). To compare snRNA-seq data from multiple libraries, the read depths were equalized across libraries by normalizing the gene-cell barcode matrices of each sample before merging. This normalization was achieved using the CellRanger aggregate procedure (44). To ensure confident mapping of reading counts for each cell library, random subsamples of the high-depth library’s reading counts were taken. The gene-cell-barcode matrices of two skin samples were then concatenated based on the number of genes detected per cell using CellRanger R (version 2.0.0) and the Seurat suite (version 2.0.0) (44). Subsequently, the concatenated matrices underwent logarithmic transformation and filtering.

### Clustering, visualization, and expression analysis

To cluster cells, Principal Component (PC) Analysis was conducted on the normalized and filtered gene-barcode matrix. The top five PCs were selected for conversion into Uniform Manifold Approximation and Projection (UMAP), enabling visualization of cell clustering in two dimensions (45). Graph-based clustering is performed to group cells based on similar expression profiles, and a nearest-neighbor graph is created without specifying the number of clusters in advance. For hierarchical clustering, we calculated the pairwise Pearson correlation between each pair of clusters based on the average expression of each gene across all cells within the clusters. To graphically represent the specific gene expression patterns, UMAP plots were generated using the Loupe Cell Browser software and Cell Ranger R (http://support.10xgenomics.com/single-cell/software/overview/welcome). To identify the pathways associated with DEGs in neutrophils, KC1, KC2, and KC5, we performed a KEGG enrichment analysis using the phyper function. False discovery rate (FDR) was used to determine the threshold of q value, and KEGG terms (FDR ≤ 0.05) were considered significantly enriched.

## Results

### Cell type composition in the skin was identified using snRNA-seq

**Figure 1:**
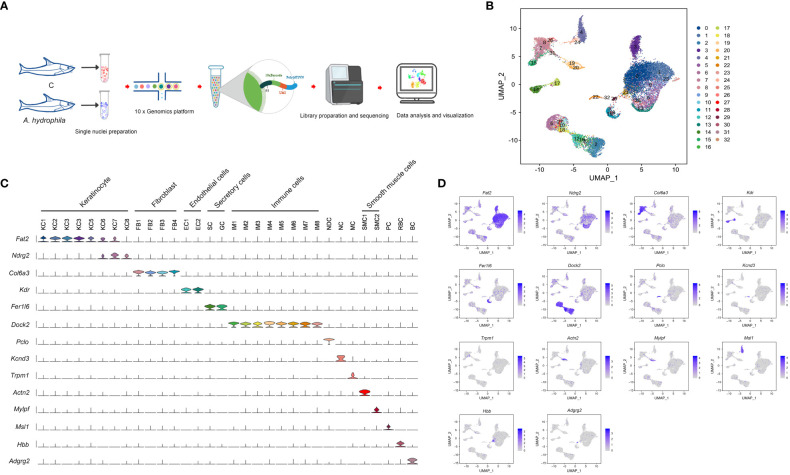
Cell sorting and identifying of cell types in the Chinese longsnout catfish skin. **(A)** The overall strategy for single nuclei RNA sequencing (snRNA-seq). **(B)** The UMAP plot for 19,581 skin cells, derived from five *A. hydrophila* infected and five healthy fishes. Different cell clusters are color-coded. **(C)** Violin plot displaying the expression of the pan or specific marker genes across the 32 identified cell clusters. Each cell cluster is labeled with different colors. **(D)** UMAP plots showing expression of pan or specific marker genes in distinct cell clusters. Blue represents cells labeled with genes. KC1 to KC8, Type 1 keratinocyte to Type 8 keratinocyte; FB1 to FB4, Type 1 Fibroblast to Type 4 Fibroblast; EC1 and EC2, Type 1 Endothelial cells and Type 2 Endothelial cells; SC, Secretory cell; GC, Goblet cell; IM1 to IM8, Type 1 immunocyte to Type 8 immunocyte; NDC, neuroendocrine cell; NC; Neurons cell; MC, Melanocyte; SMC1 and SMC2, Type 1 smooth muscle cells and Type 2 smooth muscle cells; PC, Pericyte; RBC, Red blood cell; BC, Brush cell.

Due to the observation of ulceration in fish skin caused by *A. hydrophila* infection, we conducted snRNA-seq analysis on skin samples obtained from both uninfected and infected fish. The snRNA-seq workflow consists of five main steps: sample preparation, single nuclei capture, library preparation, sequencing, and visualization (**Figure 1A**). After filtering and correction, transcriptome profiles of 19,581 single nuclei cells were obtained by analyzing the total Unique Molecular Index (UMI) counts (**Table S1**, **Figure S1**). The dataset consisted of 10,400 cells in the control group and 9,181 in the treatment group. Following UMAP dimensionality reduction and unsupervised cell clustering, 33 cell clusters were characterized and cell types were classified for all samples (**Figure 1B**; **Figures S2**, **S3**). To characterize the clusters, we performed an analysis of differentially expressed genes and inferred their putative identities using known markers (**Figures S2**, **S3**). Based on the expression of these markers, the 33 cell clusters were roughly categorized into 12 cell types, including keratinocytes, fibroblasts, and immune cells (**Figures 1C, D**; **Tables S2**, **S3**).

**Figure 2:**
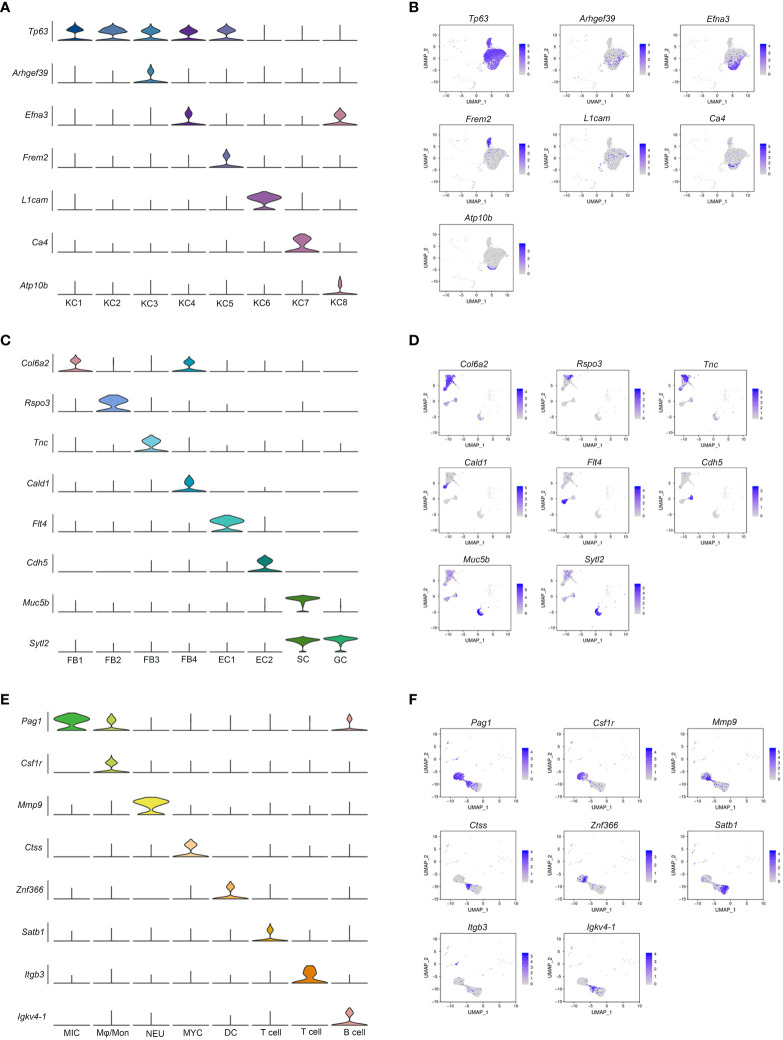
Heterogeneity of cell subpopulations in control and *A. hydrophila*-infected skin. UMAP plots and accompanying violin plots of representative genes for KC **(A, B)**; FB, EC, SC, and GC **(C, D)**; and immune-related cells **(E, F)**. The expression level is color-coded. MIC, microglial cells: Mφ/Mon, Macrophage/Monocyte; NEU, Neutrophils; MYC, Myeloid cell; DC, Dendritic cell.

Based on the expression of *fat2* and *ndrg2*, eight keratinocyte clusters were identified (KC1 - 8) (**Figures 1C, D**). KC.1 and KC.2 were highly correlated among the KC clusters, and both clusters highly expressed the KC marker genes *tp63* and *notch3* (**Figure S4**, **Table S3**), making it difficult to distinguish them by marker genes. We conclude, based on the expression of the specific marker gene (**Table S3**), that KC.1 and KC.2 are basal cells. KC.3 was identified as cycling basal cell keratinocytes with high expressions of the cycling basal cell marker genes *arhgef39*, *aspm*, *mki67*, and *diaph3* (**Figures 2A, B**; **Table S3**). KC.4 and KC8 were highly correlated and identified by some epithelial stem cell marker genes including *efna3*, *erbb3*, and *ppl*, whereas KC8 specifically expressed *atp10b*, *pttg1ip*, and *msln* (**Figures 2A, B**; **Table S3**). The KC.5 corresponded to epithelial KC cells that express *frem2*, *itgb4*, and other specific markers, whereas the KC.6 was identified as a collecting duct cell that expresses *l1cam*, *hepacam2*, and *sgk1* (**Figures 2A, B**; **Table S3**). Based on the specific expression of *ca4*, we finally identified KC.7 as a capillary cell (**Figures 2A, B**).

The elevated expression of *fer1l6*, *sytl2*, and *rasef* is characteristic of secretory cells and goblet cells, both of which have the ability to secrete substances. Nonetheless, secretory cells can be more specifically identified by the considerable expression of *muc5b*, *muc5c*, *fcgbp*, *spdef*, and *agr2*, while the *syt7* gene is distinctive to goblet cells (**Figures 1C, D**, **2C, D**; **Table S3**).

Fibroblasts (FB1-FB4) were identified by the expression of *col6a3*, *antxr1*, *col1a1*, *dcn*, and *col5a1* and were distinguished from each other through a special marker gene (**Figures 1C, D**, **2C, D**; **Table S3**). The FB1 and FB4 expressed both *col6a2* and *postn* but were distinguished by *cald1*, *itga8*, and *brinp1*. The FB3 specifically expressed *col11a1* and *tnc*, and FB2 was distinguished with the specific expression of *cald1* and *ednrb* (**Figures 2C, D**; **Table S3**).

Endothelial cells express *kdr*, *pde2a*, *fgd5*, and *ptprb*. However, they can be further distinguished based on the expression of additional markers. For instance, EC1 is characterized by the expression of *flt4*, *prox1*, *exoc3l2*, *stab2*, *vwf*, and *znf521*, while EC2 is characterized by the expression of *cdh5*, *flt1*, *podxl*, *abcg2*, *mmrn2*, *sema3g*, *ldb2*, and *arhgap31* (**Figures 1C, D**, **2C, D**; **Table S3**).

According to the correlation analysis, we discovered that the correlation between pericyte cells and two clusters of muscle cells is relatively strong (**Figure S4**). Pericytes (PC) were characterized by the expression of pericyte marker genes *msi1* and *jag1*. Two distinct clusters of muscle cells were identified based on the specific expression of muscle cell marker genes. One subset of muscle cells exhibited a high expression of the muscle marker genes *actn2*, *tnni1*, *myh6*, and *itga7*. The other subset displayed specific expressions of the muscle marker genes *mylpf* and *myh4.* Additionally, both clusters expressed the muscle cell marker gene *tnnt3* (**Figures 1C, D**; **Table S3**).

Melanocytes (MC), neuroendocrine cells (NDC), and neuronal cells (NC) are also recognized by specific marker genes. Melanocytes are characterized by the expression of several marker genes, including *trpm1*, *akap12*, *cdh7*, and *slc35f1* (**Figures 1C, D**; **Table S3**). Both neuroendocrine and neuronal cells express *kcnma1*, but they can be further differentiated by specific marker genes. Neuroendocrine cells specifically express *pclo*, *cib2*, *rimbp2*, and *kif1a*, whereas neuronal cells specifically express *kcnd3*, *stk32b*, *pcdh9*, and *cacna2d1* (**Figures 1C, D**; **Table S3**).

Seven immune-related cell types were marked by *dock2*, which further separated into microglial cells, monocytes/macrophages (Mφ/Mon), neutrophils, myeloid cell, dendritic cell, T cell, and B cell by expression of *pag1*, *csf1r*, *mmp9*, *ctss*, *znf366*, *satb1* and *itgb3*, and *igkv4-1*, respectively (**Figures 1C, D**, **2E, F**; **Table S3**). A subset of red blood cells with specific expressions of related marker genes, such as *hbb*, *epb41*, and *tfrc*, and brush cells marked by *adgrg2*, *adgrl3*, *pla2g4a*, *slitrk6*, *pigr*, and *plcb1* was subsequently identified (**Figures 1C, D**; **Table S3**).

### Expansion and differentially expressed gene analysis of keratinocyte cells in *A. hydrophila*-infected fish compared with controls

**Figure 3:**
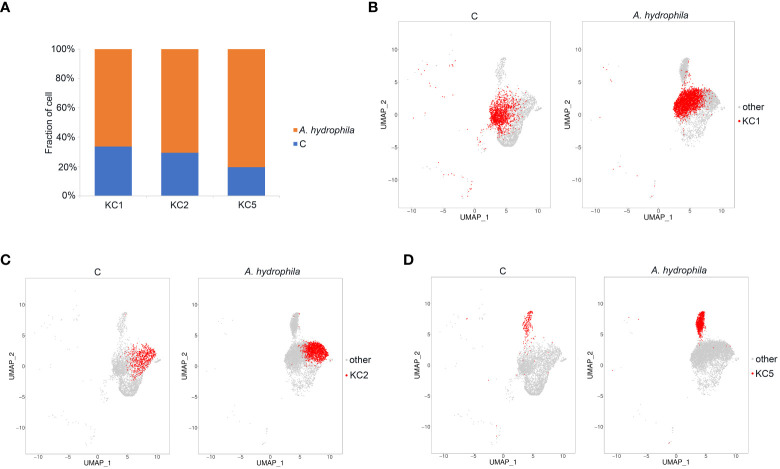
Identification of KC expansion clusters. **(A)** The proportion of cells in the three subpopulations of KC in the control and *A. hydrophila*-infected Chinese longsnout catfish. **(B-D)** The UMAP plots demonstrate the expansion of KC1, KC2, and KC5 clusters between the control group and the Chinese longsnout catfish infected with *A. hydrophila*.

**Figure 4:**
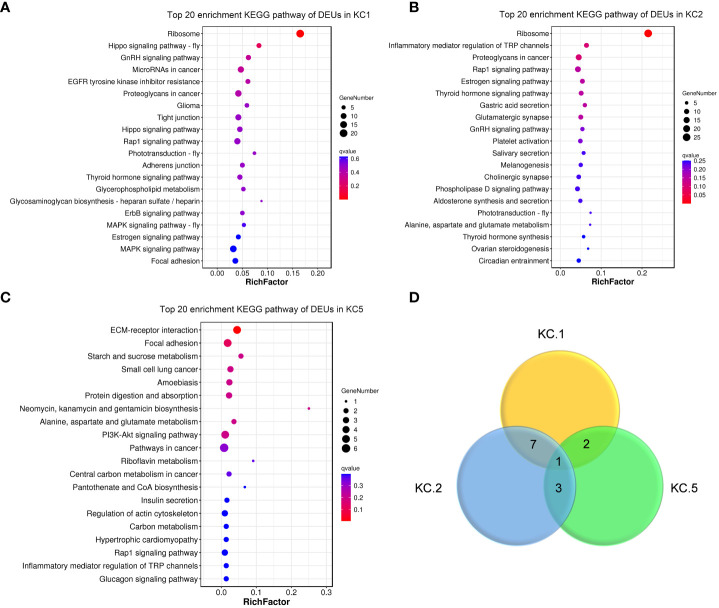
KEGG pathway enrichment analysis of DEUs among expansive KC clusters. The top 20 enriched KEGG pathways of DEUs in KC1 **(A)**, KC2 **(B)**, and KC5 **(C)**. **(D)** Venn diagram of top 20 enriched KEGG pathway in KC1, KC2, and KC5. The y-axis represents pathways, while the x-axis represents the enrichment factor. The enrichment factor is calculated as the ratio of the number of differentially expressed genes in a pathway to the total number of differentially expressed genes. The size of each representation indicates the quantity, with larger representations indicating a higher count. The color gradient ranges from red to lighter shades, with smaller values of the Q-value associated with a redder color.

Of the keratinocyte cell subgroups, cell numbers in the three clusters KC1, KC2, and KC5 were expanded after *A. hydrophila* infection (from 2.23- to 4.65-fold) (**Figures 3A–D**; **Table S4**). KC1 and KC2 had comparable expansion frequencies (2.23 folds and 2.75 folds), whereas KC5 exhibited a more pronounced increase after *A. hydrophila* infection (4.65 folds). To further investigate the transcriptomic changes of KC1, KC2, and KC5 after *A. hydrophila* infection, we compared the expression patterns of control and *A. hydrophila*-infected fish. A total of 428, 372, and 70 differentially expressed unigenes (DEUs) (probability ≥0.8 and relative change ≥2) were identified from KC1, KC2, and KC5, respectively (**Figure S5**). Our analysis revealed that these DEUs identified in our study were significantly enriched in distinct signaling pathways from the Kyoto Encyclopedia of Genes and Genomes (KEGG). Among the top 20 pathways enriched in KC1, we observed six pathways related to signal transduction, including the Hippo signaling pathway (fly), Hippo signaling pathway, Rap1 signaling pathway, ErbB signaling pathway, MAPK signaling pathway (fly), and MAPK signaling pathway. Additionally, four pathways were associated with organismal systems, including three pathways related to the endocrine system (GnRH signaling pathway, Thyroid hormone signaling pathway, and Estrogen signaling pathway), and one pathway related to the Sensory system (Phototransduction - fly). Furthermore, three pathways involved in Cellular Processes were identified (Tight junction, Adherens junction, and Focal adhesion), along with two pathways associated with Metabolism (Glycerophospholipid metabolism and Glycosaminoglycan biosynthesis - heparan sulfate/heparin) (**Figure 4A**). The results indicate that KC1 is mainly involved in environmental signal transduction function. However, compared to KC1, the top 20 enriched pathways for the DEUs in KC2 are mainly involved in organismal systems (including 15 pathways). These comprised seven pathways for the endocrine system (Estrogen signaling pathway, Thyroid hormone signaling pathway, GnRH signaling pathway, Melanogenesis, Aldosterone synthesis and secretion, Thyroid hormone synthesis, and Ovarian steroidogenesis), two pathways for the sensory system (Inflammatory mediator regulation of TRP channels and Amino acid metabolism), two pathways involved in the nervous system (Glutamatergic synapse and Cholinergic synapse), two pathways involved in the Digestive system (Gastric acid secretion and Salivary secretion), one pathway involved in the immune system (Platelet activation), and one pathway related to the environmental adaptation (Circadian entrainment) (**Figure 4B**). The results indicate that KC2 was mainly involved in endocrine function. Compared to the above two, KC5 was involved in more metabolic-related pathways in the top 20 pathways. These included the metabolism of starch and sucrose; the biosynthesis of neomycin, kanamycin, and gentamicin; the metabolism of Alanine, aspartate, and glutamate; the metabolism of riboflavin; the biosynthesis of pantothenate and coenzyme A; and the metabolism of carbon (**Figure 4C**). The results demonstrate the significance of KC5 in metabolism. Furthermore, most of the top 20 signaling pathways are not shared by both KC1 and KC2. This indicates that they are distinct cell types (**Figure 4D**).

### Expansion and differential gene expression analysis of neutrophils in *A. hydrophila*-infected versus uninfected individuals

**Figure 5:**
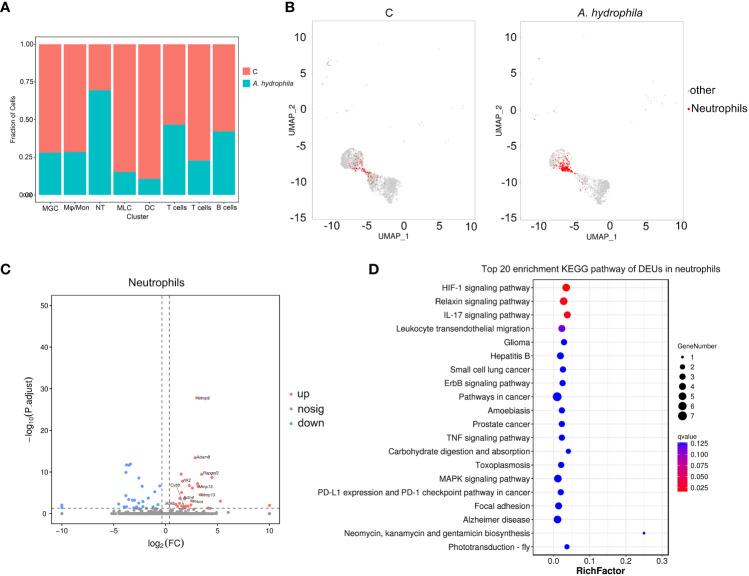
Immune response analysis of immune cell subsets. **(A)** Proportion of immune cell subsets in the control and *A. hydrophila*-infected Chinese longsnout catfish. **(B)** The UMAP plots align the neutrophil clusters between the control and *A. hydrophila*-infected Chinese longsnout catfish. **(C)** The volcano plot of DEGs from neutrophil cells in the comparison of the control and *A. hydrophila*-infected fish. Each red or blue dot represents an individual gene and grey dots represent non-significant genes. The example genes were labeled with their corresponding names. **(D)** The top 20 enriched KEGG pathway of DEUs in neutrophils.

In addition, we determined the relative proportion of each immune cell type in all samples and found that the proportion of neutrophils was significantly higher in *A. hydrophila*-infected samples than in samples from the control group (**Figures 5A, B**; **Table S4**). Through inter-group differential analysis, we identified 55 DEUs, among which multiple inflammation-related genes, including *mmp9*, *mmp13*, and *actn4*, showed upregulated expressions (**Figure 5C**). Pathway enrichment analysis showed neutrophils with significant enrichment of terms of the IL-17 signaling pathway and Leukocyte transendothelial migration (**Figure 5D**), which indicated that the invasion of *A. hydrophila* leads to the occurrence of skin inflammation. The above results suggested that neutrophils may play an important role during *A. hydrophila* infection.

## Discussion

*A. hydrophila* is a common fish pathogen that can cause systemic skin ulcers in fish (46). In order to comprehend the pathogenesis of an *A. hydrophila* infection, it is essential to identify the skin regions and cell types targeted by the bacteria. In this study, snRNA-seq was utilized to identify 33 transcriptionally distinct cell subtypes in the *L. longirostris* skin, including eight KCs, four FBs, two ECs, and eight IMs (**Figure 1**). Additionally, we discovered that *A. hydrophila* infection led to a significant increase in three KC clusters and one neutrophil cluster. Pathway enrichment analysis revealed three distinct KC clusters exhibiting enrichment in the following terms: signal transduction, endocrine system, and metabolism, respectively. Additionally, neutrophils showed enrichment in terms related to inflammatory response. These findings present a comprehensive overview of skin cell types based on their transcriptional characteristics and provide valuable insights into the identification of cell types involved in the antibacterial response.

Previously, researchers conducted a census of cell types in human and mouse skin (11, 47–50). Despite the significant role of fish in vertebrate evolution, the knowledge of the specific cell types present in fish skin remains limited. To address this knowledge gap, we conducted this study using juvenile Chinese longsnout catfish (*Leiocassis longirostris*) infected with *A. hydrophila*. Through the single nuclei cell collection, we successfully sequenced a total of 10,400 cells from the control group and 9,181 cells from the *A. hydrophila*-infected group of fish skin. In addition, we have successfully identified cell type-specific markers, which will facilitate a clear and unequivocal definition of cell types. This achievement sets the stage for the future development of genetic tools that enable precise labeling and manipulation of specific cell types. This capability is crucial for analyzing the specific functions of each cell type within the intricate skin region. In mammals, cells clustered in different skin regions have been identified based on their distinct expression of marker genes. These cell types include KC, FB, EC, and PC, as well as nerve-related and immunity-related cells (11, 51). Previously, the types of cells in fish skin were unknown. However, by utilizing the marker genes found in skin clusters in mammals as a reference, we were able to identify 12 distinct skin cell types in the Chinese longsnout catfish, including keratinocytes (*fat2* + *ndrg2*) (52), fibroblast (*col6a3*+ *dcn*) (53, 54), endothelial cells (*ptprb*+ *pde2a*) (55, 56), secretory cells (*muc5b* + *sytl2* + *slitrk6*) (52, 57, 58), smooth muscle cells (*tnnt3*+ *tnni1*+ *mylpf*) (59–61), immune-related cells (*dock2* + *fyb1*) (62, 63), and neurons-related cells (*kcnma1*+ *pclo*) (52, 64). This indicated that the skin cell divisions of mammals also apply to fish (47, 63, 65). In most cases, a skin cluster was defined by the combined expression of marker genes. However, it is worth noting that the majority of these clusters contained subtype-specific genes that were unique and specific to that particular cluster (**Figures 1C**, **2**; **Table S3**). For instance, specific marker genes to differentiate between KC1 and KC2 have not yet been identified. Furthermore, certain genes were found to be exclusively expressed in specific types of skin cells. *Fat2* was only expressed in KC cells, such as KC1, KC2, KC3, KC4, KC5, KC6, and KC7 (**Figures 1C, D**). In addition, although specific marker genes that can readily distinguish between KC1 and KC2 have yet to be discovered, pathway analysis has indicated that among the top 20 pathways, KC1 is primarily involved in signal transduction, whereas KC2 is primarily associated with endocrine function (**Figures 4A, B**). This finding suggests that KC1 and KC2 remain distinct and belong to separate cell clusters. These results demonstrate that our unbiased snRNA-seq analyses effectively identified different cell types and their corresponding transcriptional characteristics in the skin.

The skin of fish plays a crucial role in the innate immune responses, especially in combating bacterial infections. Numerous studies have documented the characteristics of the skin immune response in fish following bacterial infection (1, 2, 66–68), which has contributed to our understanding of the potential pathogenesis of *A. hydrophila* infection. However, it is challenging to obtain a comprehensive understanding of the cellular and molecular immune responses in fish infected with *A. hydrophila*. In this study, we examined the immunological response in Chinese longsnout catfish infected with *A. hydrophila* at a single-cell resolution. The purpose was to identify the specific region responsible for antibacterial immunity in the infected fish. In human inflammatory skin diseases, it is well-known that there are often phenomena such as excessive proliferation of epidermal keratinocytes and abnormal keratinization or terminal differentiation of the epidermis (69–71). The number of KC1, KC2, and KC5 cells in infected fish increased by 2.23, 2.72, and 4.65 times, respectively, according to the results of this study. However, a limitation of this conclusion is the low sample size. In addition, the infection caused by *A. hydrophila* resulted in significant changes in the transcription of epithelial proliferation and migration-related genes, including *tp63*, *fat2*, and *Itgb4*, when compared to control fish. Additionally, it was observed that the Rap1 signaling pathway was enriched into the top 20 pathways for the DEUs within the three KC clusters that exhibited expansion. Moreover, the inflammatory mediator regulation of the TRP channels pathway was also enriched in the top 20 enriched pathways for the DEUs in KC2 and KC5 respectively (**Figures 4B, C**). These results indicate that bacterial invasion may trigger an inflammatory response in fish skin, ultimately leading to epithelial cell proliferation. In addition, six metabolism-related pathways were enriched into the top 20 KEGG pathways for the DEUs of KC5, and these DEUs are all down-regulated in their expression (**Figure S5D**). This suggests that KC5 may primarily influence skin metabolism in response to bacterial infection.

Although multiple studies have confirmed the importance of fish skin in innate immunity, the specific immune mechanism and cell types involved are currently unclear (26, 72, 73). Single-cell RNA sequencing offers a more direct, reliable, and detailed analysis of immune subpopulations, in comparison to traditional large-scale transcriptome studies that rely on open chromatin analysis (11). For example, we identified multiple types of immune cells in the skin of Chinese longsnout catfish including T cell (*satb1*+ *itgb3*) (74, 75), B cell (*igkv4-1*, *igll5*) (76, 77), DC (*shtn1*+ *znf366*) (78), Mo/MΦ (*csf1r* + *slc2a6*) (76, 79), and neutrophils (*mmp9*+ *adam8*) (74, 80), and detected subtlety to the expansion of neutrophils in infected fish (**Figures 5A, B**). Neutrophils are a critical component of the immune system that helps the fish body fight infections by attacking and destroying harmful bacteria and other invaders (81–84). Here, we also found that the IL-17 signaling pathway and Leukocyte transendothelial migration pathway were significantly enriched for the DEUs in neutrophils. The results confirm the role of neutrophils in the immune reaction after bacterial infection in Chinese longsnout catfish.

## Conclusions

Our research represents the first comprehensive comparative transcriptomic analysis of non-lesional skin from Chinese longsnout catfish, comparing infected samples with healthy controls. Moreover, this analysis has provided valuable insights into the complex interplay between different cell types and the changes in gene expressions associated with *A. hydrophila* infections. By elucidating these mechanisms, we contribute to a greater understanding of host-pathogen interactions in fish skin and pave the way for future research in this area. Using snRNA-seq analysis, we identified 12 cell types, including KC, FB, EC, and IM. Numerous new pathogenesis-related phenomena, including epidermal hyperplasia and keratinocyte differentiation disruption, were triggered by *A. hydrophila* infection in Chinese longsnout catfish. Based on the increase in cell proportion and KEGG pathway enrichment analysis, we concluded that neutrophils may be the key cell type required for *A. hydrophila* eradication. This study resulted in a detailed cellular atlas of fish skin and a greater comprehension of how *A. hydrophila* attacks the skin and how the skin resists bacteria.

## Data availability statement

The original contributions presented in the study are publicly available. This data can be found here: https://www.ncbi.nlm.nih.gov/bioproject/PRJNA991636.

## Ethics statement

The animal study was approved by the Animal Care and Use Committee of the Fishery Institute of the Sichuan Academy of Agricultural Sciences (20170226001A). The study was conducted in accordance with the local legislation and institutional requirements.

## Author contributions

CM: Writing – original draft, Writing – review & editing. LZ: Investigation, Methodology, Writing – review & editing. HZ: Formal Analysis, Validation, Writing – review & editing. ZH: Methodology, Resources, Writing – original draft. YD: Conceptualization, Supervision, Writing – review & editing. ZZ: Software, Visualization, Writing – original draft. HK: Investigation, Resources, Writing – review & editing. JD: Funding acquisition, Resources, Writing – review & editing. QL: Project administration, Resources, Supervision, Writing – review & editing. JZ: Funding acquisition, Project administration, Supervision, Writing – review & editing.

## Supplementary material

Supplementary Figure 1 The distribution of basic information of each group cell and scatter diagram of the basic information of each group after filtration. **(A)** The distribution of basic cell information in each group before filtering is shown in the figure. The scatter plot in the figure is colored based on cell type, with red representing multicellular cells and black representing single cells. **(B)** The distribution of basic cell information in each group after filtering. The y-axis in the left graph represents the number of genes detected in individual cells of the two samples, while the y-axis in the right graph represents the number of unique molecular identifiers (UMIs) detected in individual cells of the two samples. **(C)** Scatter plot of the distribution of basic cell information. The scatter plot represents cells from different groups with different colors. The x-axis represents the number of unique molecular identifiers (UMIs) detected in individual cells of the two samples, while the y-axis represents the number of genes detected in individual cells of the samples. The number above represents the Pearson correlation coefficient between the aforementioned two variables.

Supplementary Figure 2 The heatmap visually displays the differential expression of cell type-specific genes across the 33 clusters. Each column represents an individual cell, and each row represents an individual gene. The expression level of a gene in different cells is indicated by various colors. Higher expression levels are represented by yellow colors, while lower expression levels are represented by purple colors.

Supplementary Figure 3 Bubble chart showing the cell type-specific genes that are differentially expressed across the 33 clusters. Bubble sizes represent the proportion of cells expressing the marker gene within a specific subpopulation, with larger bubbles indicating a higher proportion. Bubble colors represent the average expression abundance of the marker gene within the cell subpopulation, with darker colors indicating higher average expression levels in that subpopulation.

Supplementary Figure 4 C The correlation heatmap of 33 clusters. The heatmap visually represents the Pearson correlation across the 33 clusters. Each column or row represents a cell cluster. The correlation coefficients between different cell clusters are represented by various colors. Higher correlations are represented by red colors, while lower correlations are represented by blue colors.

Supplementary Figure 5 Enrichment circle plot of expanded cell clusters based on Gene Ontology (GO) analysis. First circle: The top 20 enriched GO terms, with the number of genes as the coordinate axis outside the circle. Different colors represent different Ontologies; Second circle: Number of background genes associated with the GO term and Q-value. The longer the bar, the more genes associated, and the redder the color, the smaller the Q-value; Third circle: Bar chart showing the ratio of upregulated to downregulated genes, with deep purple representing the upregulated gene ratio and light purple representing the downregulated gene ratio. Specific values are shown below; Fourth circle: Rich Factor values for each GO term (number of differentially expressed genes divided by the total number of genes), with background grid lines, where each grid represents 0.1.
