# Supplementary figures and images for: Single-nuclei RNA-seq reveals skin cell responses to Aeromonas hydrophila infection in Chinese longsnout catfish Leiocassis longirostris
Source: Front Immunol. 2023 Oct 16;14:1271466. doi: 10.3389/fimmu.2023.1271466 (PMC10613986; doi:10.3389/fimmu.2023.1271466)

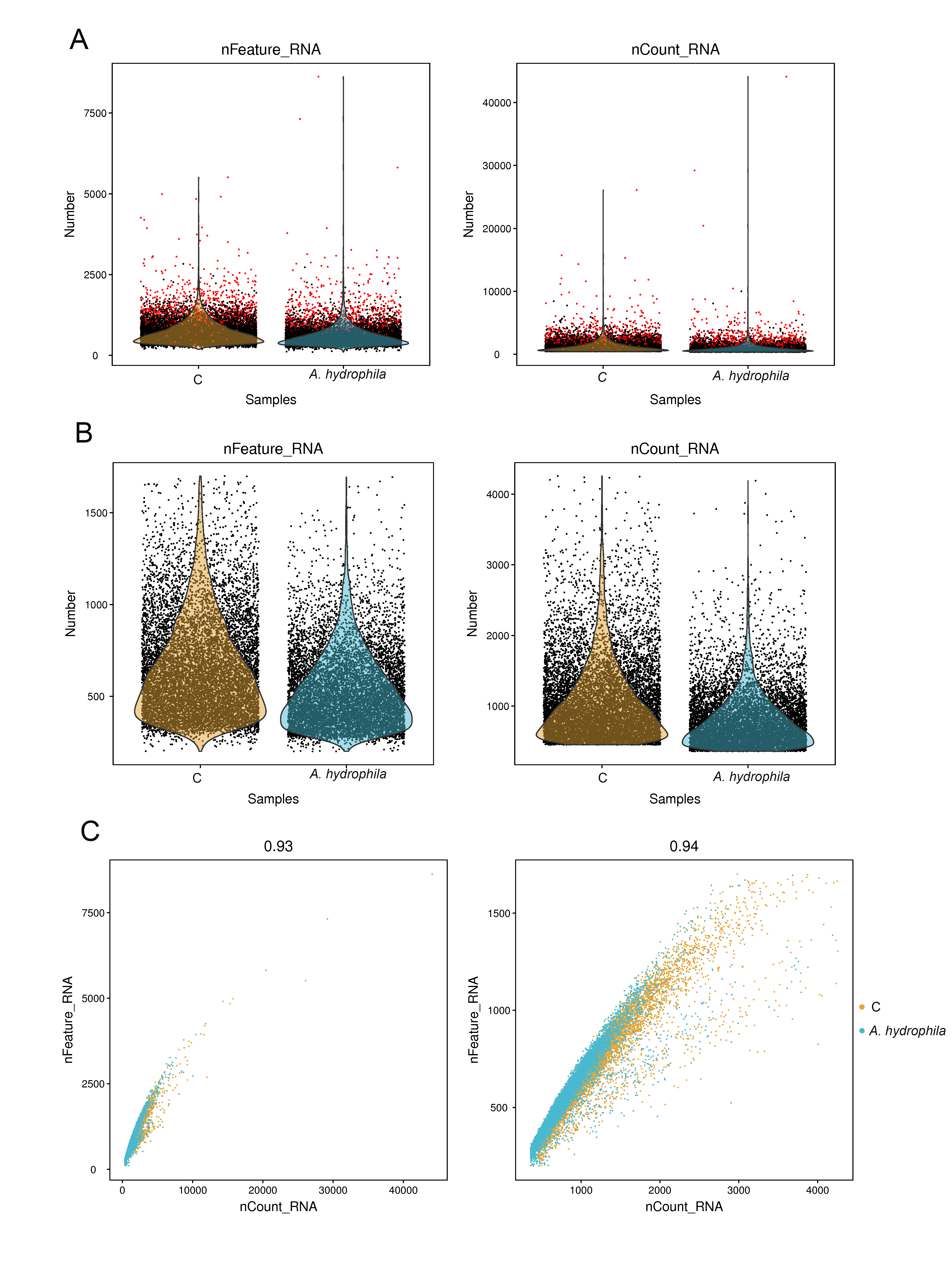

Supplement: Supplementary Figure 1 — The distribution of basic information of each group cell and scatter diagram of the basic information of each group after filtration. (A) The distribution of basic cell information in each group before filtering is shown in the figure. The scatter plot in the figure is colored based on cell type, with red representing multicellular cells and black representing single cells. (B) The distribution of basic cell information in each group after filtering. The y-axis in the left graph represents the number of genes detected in individual cells of the two samples, while the y-axis in the right graph represents the number of unique molecular identifiers (UMIs) detected in individual cells of the two samples. (C) Scatter plot of the distribution of basic cell information. The scatter plot represents cells from different groups with different colors. The x-axis represents the number of unique molecular identifiers (UMIs) detected in individual cells of the two samples, while the y-axis represents the number of genes detected in individual cells of the samples. The number above represents the Pearson correlation coefficient between the aforementioned two variables. [file Image_1.tif]

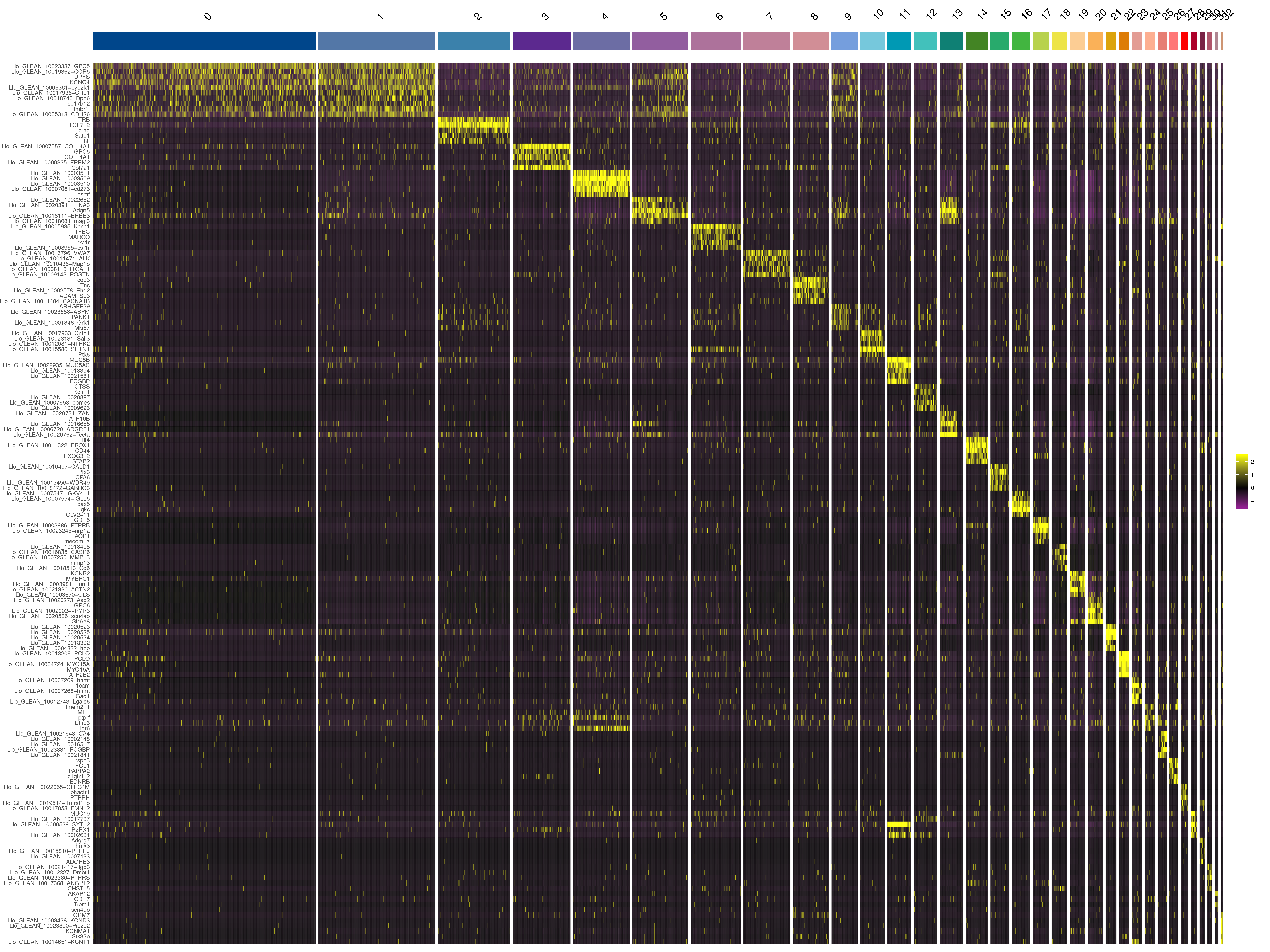

Supplement: Supplementary Figure 2 — The heatmap visually displays the differential expression of cell type-specific genes across the 33 clusters. Each column represents an individual cell, and each row represents an individual gene. The expression level of a gene in different cells is indicated by various colors. Higher expression levels are represented by yellow colors, while lower expression levels are represented by purple colors. [file Image_2.png]

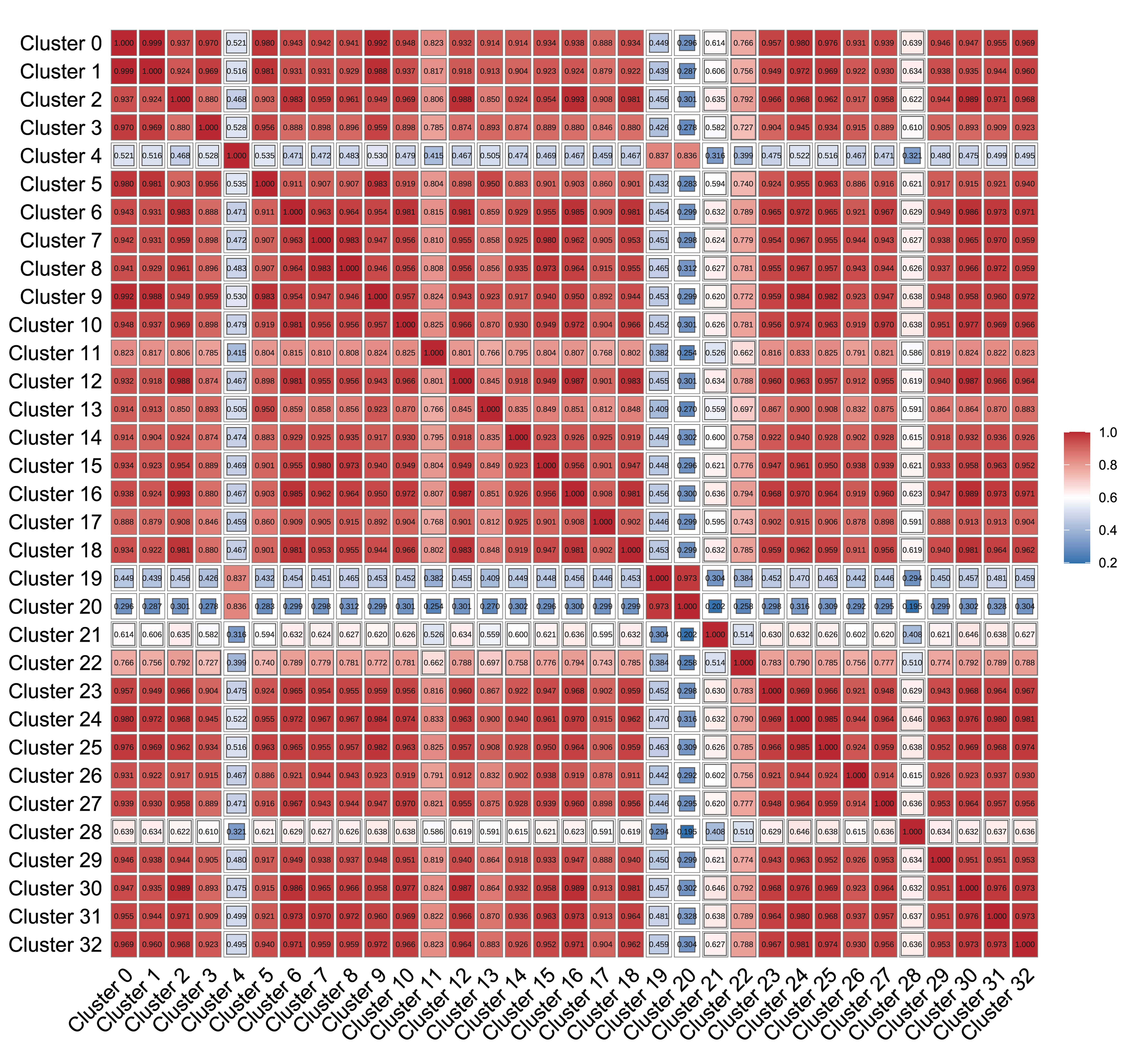

Supplement: Supplementary Figure 4 — C The correlation heatmap of 33 clusters. The heatmap visually represents the Pearson correlation across the 33 clusters. Each column or row represents a cell cluster. The correlation coefficients between different cell clusters are represented by various colors. Higher correlations are represented by red colors, while lower correlations are represented by blue colors. [file Image_4.tif]

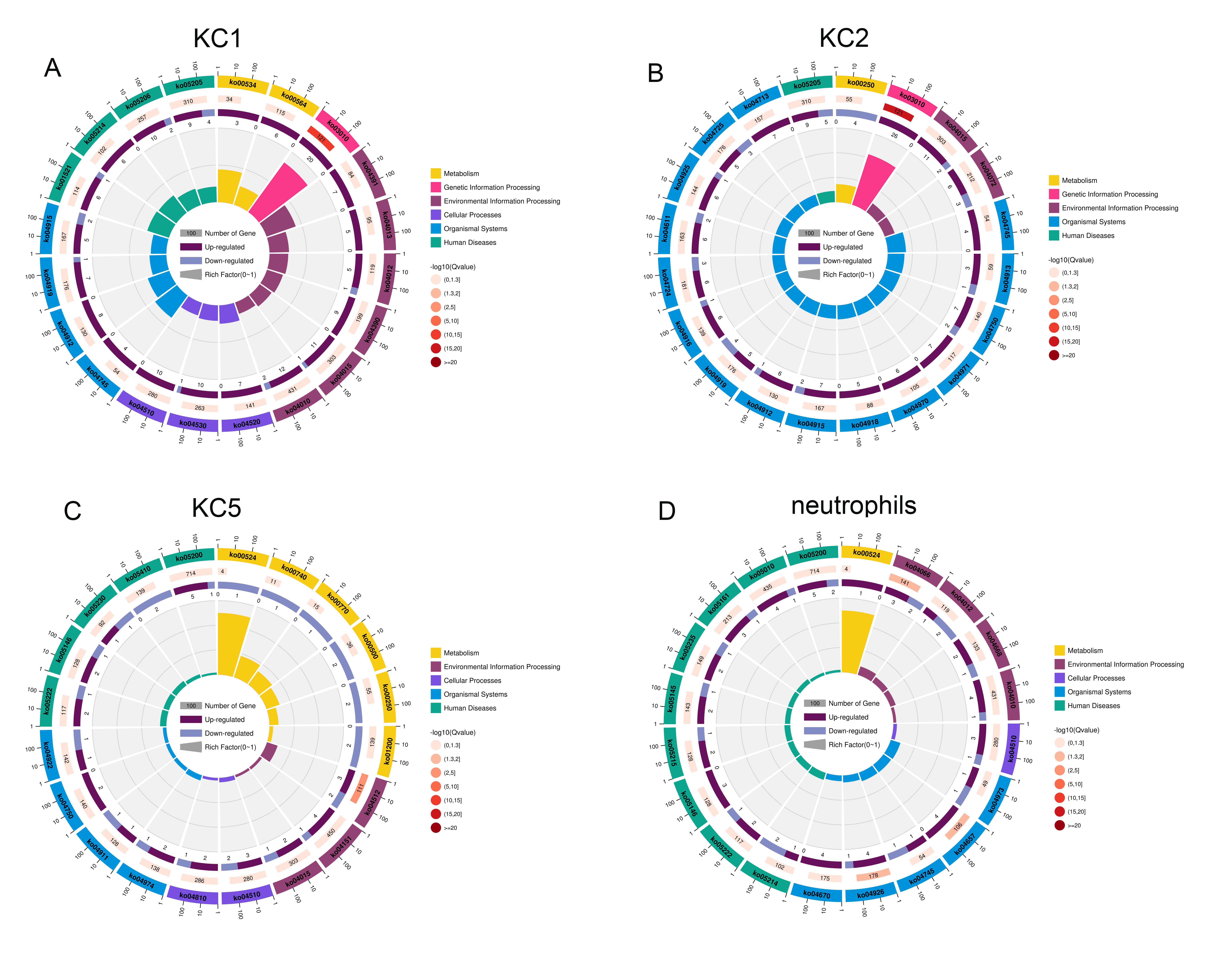

Supplement: Supplementary Figure 5 — Enrichment circle plot of expanded cell clusters based on Gene Ontology (GO) analysis. First circle: The top 20 enriched GO terms, with the number of genes as the coordinate axis outside the circle. Different colors represent different Ontologies; Second circle: Number of background genes associated with the GO term and Q-value. The longer the bar, the more genes associated, and the redder the color, the smaller the Q-value; Third circle: Bar chart showing the ratio of upregulated to downregulated genes, with deep purple representing the upregulated gene ratio and light purple representing the downregulated gene ratio. Specific values are shown below; Fourth circle: Rich Factor values for each GO term (number of differentially expressed genes divided by the total number of genes), with background grid lines, where each grid represents 0.1. [file Image_5.tif]
